# Preoperative monocyte-to-albumin ratio predicts overall survival after hepatectomy for hepatocellular carcinoma: a single-center retrospective study

**DOI:** 10.1186/s12957-026-04429-w

**Published:** 2026-06-06

**Authors:** Xin Jiang, Lu-Yun Zhang, Jia-Peng Liao, Xiong Tang, Gao-Min Liu, Ji-Wei Xu

**Affiliations:** 1 Department of Hepatobiliary Surgery, Meizhou People’s Hospital, No. 63 Huangtang Road, Meizhou, 514000 China; 2 China Meizhou Clinical Institute of Shantou University Medical College, No. 63 Huangtang Road, Meizhou, 514000 China

**Keywords:** Hepatocellular carcinoma, Hepatectomy, Monocyte-to-albumin ratio, Advanced lung cancer inflammation index, Survival, Nomogram

## Abstract

**Background:**

Hepatocellular carcinoma (HCC) is a major cause of cancer-related death worldwide. Hepatectomy is a potentially curative option for selected patients, but postoperative recurrence and death remain frequent. There is a need for simple, low-cost indices that can be measured before surgery to improve risk stratification. The monocyte-to-albumin ratio (MAR) reflects systemic inflammation and nutritional status. This study evaluated whether preoperative monocyte-to-albumin ratio predicts overall survival (OS) in patients with hepatocellular carcinoma undergoing hepatectomy and aimed to construct a prognostic nomogram incorporating this ratio for individualized survival estimation.

**Methods:**

We retrospectively analyzed 539 consecutive patients with pathologically confirmed hepatocellular carcinoma who underwent curative-intent hepatectomy at Meizhou People’s Hospital between May 2011 and July 2023. Clinical characteristics, laboratory tests, tumor features, and survival data were obtained from medical records. Monocyte-to-albumin ratio was calculated as monocyte count × 1000 / albumin (g/dL). The optimal cut-off value was determined using R software. Overall survival was estimated by the Kaplan–Meier method and compared between groups using log-rank tests. Cox proportional hazards regression was used to identify independent prognostic factors. A prognostic nomogram was developed based on multivariable Cox analysis and internally assessed using the concordance index(C-index), time-dependent receiver operating characteristic curves(ROC), and calibration plots.

**Results:**

Patients were classified into high and low monocyte-to-albumin ratio groups according to the optimal cut-off. Those with a high ratio had significantly shorter overall survival than those with a low ratio (log-rank *p* < 0.001). In multivariable Cox analysis, a lower monocyte-to-albumin ratio was independently associated with better overall survival after adjustment for standard clinicopathological variables (hazard ratio 0.58, 95% confidence interval 0.42–0.82, *p* < 0.001). The nomogram that combined monocyte-to-albumin ratio with other independent factors showed good discrimination and calibration, and provided higher prognostic accuracy than single predictors.

**Conclusions:**

Preoperative MAR is an independent prognostic marker of OS in patients with HCC undergoing hepatectomy. A nomogram integrating this ratio with routine clinicopathological factors may offer a practical tool for individualized risk assessment and postoperative management.

**Supplementary Information:**

The online version contains supplementary material available at 10.1186/s12957-026-04429-w.

## Introduction

Hepatocellular carcinoma is a common malignancy worldwide and remains a major contributor to cancer-related mortality [1]. In China, liver cancer continues to rank among the leading causes of cancer death [2]. For patients who are suitable surgical candidates, hepatectomy and liver transplantation are still regarded as the main curative options and can provide meaningful long-term survival [3]. However, the prognosis after surgery is often compromised by a high rate of tumor recurrence; up to 70% of patients experience relapse within five years, and earlier recurrence is frequently associated with an unfavorable outcome [4]. In this context, there is a clear need for sensitive and practical prognostic markers that can help stratify postoperative risk and guide subsequent clinical decision-making.

The monocyte-to-albumin ratio has recently been proposed as an index that reflects both the systemic inflammatory response and nutritional status [5]. Albumin, synthesized mainly in the liver, is an established indicator of hepatic synthetic function and has been repeatedly associated with the severity and prognosis of chronic liver diseases and liver malignancies [6–10]. Previous work has shown that elevated MAR is linked to worse survival, particularly in patients with hepatitis B virus–associated decompensated cirrhosis, in whom MAR was identified as an independent predictor of short-term mortality [11].

Another composite index, the Advanced Lung Cancer Inflammation Index (ALI), also integrates inflammatory and nutritional parameters and has been applied beyond lung cancer [12, 13]. Liu et al. demonstrated that ALI could serve as a prognostic marker for overall survival and cancer-specific survival in HCC patients undergoing hepatectomy, particularly for long-term outcomes [14]. Despite these findings, data focusing specifically on the prognostic impact of MAR in high-risk HCC populations after hepatectomy are still scarce. Clarifying the role of MAR in this setting may help refine perioperative risk assessment and tailor postoperative surveillance and adjuvant strategies.

Therefore, in the present study, we investigated the prognostic significance of preoperative MAR in patients with HCC who underwent surgical resection. Our objective was to identify patients at increased risk of poor survival and to provide evidence to support individualized postoperative management. This study was designed and reported in accordance with the STROBE guidelines.

## Methods

### Study population

We conducted a retrospective cohort study of patients who underwent liver resection at Meizhou People’s Hospital between May 2011 and July 2023. A total of 539 consecutive individuals were included. All enrolled patients had complete preoperative laboratory tests and clinical records available for analysis. The study protocol followed the ethical principles of the Declaration of Helsinki, and, given the retrospective nature of the work, the requirement for informed consent was waived by the institutional ethics committee.

### Inclusion and exclusion criteria

Patients were eligible for inclusion if they met all of the following criteria:first-time diagnosis of liver cancer with no prior antitumor treatment before surgery; istopathological confirmation of HCC; hepatectomy performed as the initial and primary treatment modality; complete clinicopathological and follow-up data available.

Patients were excluded if they met any of the following criteria:overall survival or recurrence-free survival of less than 90 days; liver malignancies other than HCC; a documented history of other malignant tumors;palliative or non-curative hepatectomy;Patients with missing data for key variables.

### Data collection

All variables were obtained from the tumor registry database of Meizhou People’s Hospital. The collected information included:demographic characteristics: age and sex;anthropometric measurements: body weight, height, and body mass index (BMI); comorbidities: presence of hypertension and/or diabetes; preoperative laboratory findings (blood samples were collected within one week before surgery): complete blood count, coagulation parameters, liver and renal function tests, myocardial enzyme profile, lipid profile, and alpha-fetoprotein (AFP); liver status: presence or absence of cirrhosis;tumor-related characteristics: tumor number, histological grade, capsule formation, vascular invasion, and microvascular invasion (MVI); outcome data: dates of surgery, last follow-up, and death.

Tumor staging was assigned according to the 8th edition of the American Joint Committee on Cancer (AJCC) TNM classification system. Follow-up was continued until July 7, 2023. OS was defined as the time interval from the date of hepatectomy to death from any cause or to the last documented follow-up for surviving patients.

### Calculation

The following indices were calculated using standard formulas:

$$\mathrm{MAR}=\mathrm{monocyte}\;\mathrm{count}\times1000/\mathrm{albumin}\;(\mathrm g/\mathrm{dL})$$

$$\mathrm{BMI}=\mathrm{weight}\;(\mathrm{kg})/\mathrm{height}^2\;(\mathrm m^2)$$

$$\mathrm{ALI}=\mathrm{BMI}\times\mathrm{albumin}\;(\mathrm g/\mathrm{dL})/\mathrm{absolute}\;\mathrm{neutrophil}\;\mathrm{count}/\mathrm{absolute}\;\mathrm{lymphocyte}\;\mathrm{count})$$

### Statistical analysis

All statistical analyses were performed using R software (version 4.4.3). The optimal cut-off value for MAR was determined with the “survminer” package (version 0.5.0). Continuous variables were expressed as mean ± standard deviation or median with interquartile range, as appropriate, and compared between groups using either independent-samples t tests or Mann–Whitney U tests. Categorical variables were summarized as counts and percentages and compared using Pearson’s chi-squared test or Fisher’s exact test, as appropriate. Survival curves were constructed by the Kaplan–Meier method, and differences between groups were assessed with the log-rank test. A two-sided *p* value < 0.05 was considered to indicate statistical significance.

To examine the functional form of the association between continuous preoperative MAR and overall survival, we employed restricted cubic splines (RCS) with four knots. This nonparametric approach allows for flexible modeling of potential nonlinear relationships without imposing linearity constraints. The linearity assumption was formally tested by comparing the RCS model to a linear Cox model containing MAR as a continuous linear term using the likelihood ratio test. Model fit was further evaluated using the Akaike Information Criterion (AIC). The RCS analysis was implemented using the “rms” package (version 8.0–0) in R software.

### Construction and internal validation of the prognostic nomogram

A prognostic nomogram was developed based on all variables that were identified as independent predictors of OS in multivariable Cox regression analysis. The calibration of the nomogram was examined using calibration plots generated by bootstrap resampling with 1000 iterations, comparing predicted survival probabilities with observed outcomes. Discriminative ability was quantified by the C-index and ROC curves. Clinical utility and net benefit across a range of threshold probabilities were evaluated using decision curve analysis (DCA).

## Results

### Clinic-pathological characteristics

**Fig. 1:**
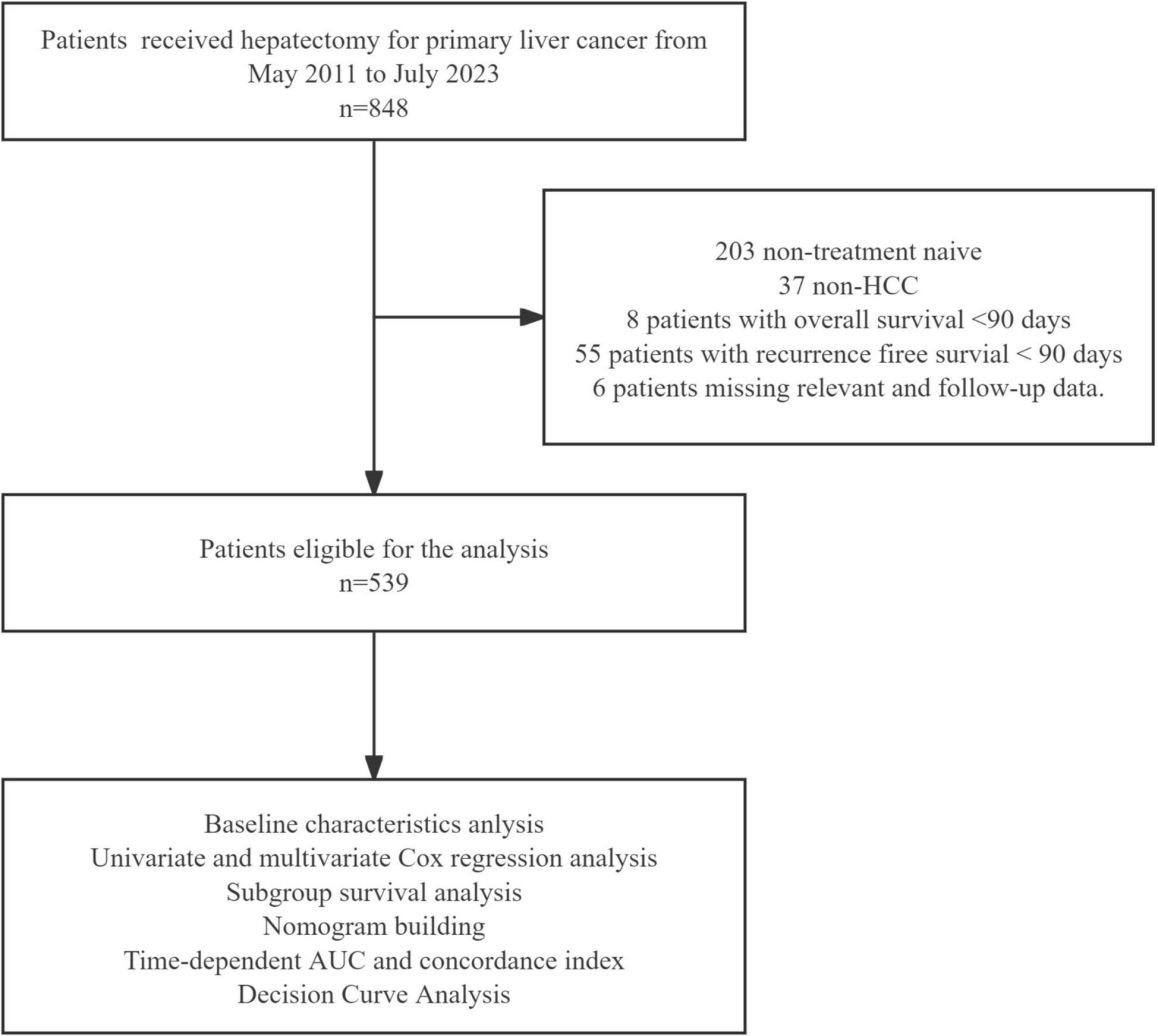
The flow chart of the study

The patient selection process is illustrated in Fig. 1. A total of 539 patients were finally included, comprising 474 men (87.9%) and 65 women (12.1%), with a median age of 59 years (range, 23–85 years). According to the AJCC 8th edition, 403 patients (74.77%) were classified as stage I–II and 136 (25.23%) as stage III–IV. The median follow-up duration was 38.1 months (range, 3.1–144.9 months). The median [IQR] preoperative MAR was 123.84 [89.99–169.70]. Using the “survminer” package, an optimal MAR cut-off of 145.7726 was identified, based on which patients were stratified into a low-MAR group (*n* = 338) and a high-MAR group (*n* = 201). The detailed baseline clinicopathological features of the cohort and of the two MAR subgroups are presented in Table 1.

**Table 1:** Baseline characteristics of included patients

Characteristics	Level	MAR^high^ (*N* = 201)	MAR^low^ (*N* = 338)	**p**	ALI^high^ (*N* = 372)	ALI^low^ (*N* = 167)	**p**
Age (mean (SD))		58.24 (11.62)	57.73 (11.03)	0.611	58.14 (10.73)	57.44 (12.35)	0.501
Gender (%)	FEMALE	20 (10.0)	45 (13.3)	0.306	46 (12.4)	19 (11.4)	0.855
	MALE	181 (90.0)	293 (86.7)		326 (87.6)	148 (88.6)	
BMI (mean (SD))		22.59 (3.81)	22.86 (3.34)	0.4	23.40 (3.61)	21.33 (2.85)	**< 0.001**
Hypertension (%)	No	164 (81.6)	273 (80.8)	0.903	293 (78.8)	144 (86.2)	0.054
	Yes	37 (18.4)	65 (19.2)		79 (21.2)	23 (13.8)	
Diabetes (%)	No	172 (85.6)	285 (84.3)	0.789	313 (84.1)	144 (86.2)	0.621
	Yes	29 (14.4)	53 (15.7)		59 (15.9)	23 (13.8)	
HBsAg (%)	Negative	44 (21.9)	54 (16.0)	0.108	71 (19.1)	27 (16.2)	0.489
	Positive	157 (78.1)	284 (84.0)		301 (80.9)	140 (83.8)	
Cirrhosis (%)	No	59 (29.4)	100 (29.6)	1	106 (28.5)	53 (31.7)	0.509
	Yes	142 (70.6)	238 (70.4)		266 (71.5)	114 (68.3)	
Portal_hypertension (%)	No	169 (84.1)	256 (75.7)	**0.029**	298 (80.1)	127 (76.0)	0.34
	Yes	32 (15.9)	82 (24.3)		74 (19.9)	40 (24.0)	
AFP (mean (SD))		2283.12 (5443.45)	2567.04 (6330.04)	0.596	2279.30 (5944.04)	2866.26 (6156.61)	0.295
Glu (mean (SD))		5.91 (2.40)	5.83 (2.38)	0.707	5.71 (2.21)	6.20 (2.72)	**0.028**
TG (mean (SD))		1.21 (0.68)	1.24 (1.23)	0.771	1.34 (1.22)	0.98 (0.46)	**< 0.001**
TC (mean (SD))		4.69 (1.27)	4.84 (1.38)	0.219	4.92 (1.38)	4.50 (1.20)	**0.001**
HDLC (mean (SD))		1.45 (0.71)	1.47 (0.67)	0.798	1.47 (0.72)	1.43 (0.61)	0.44
LDLC (mean (SD))		2.55 (0.93)	2.65 (0.89)	0.2	2.67 (0.91)	2.48 (0.90)	**0.02**
ALB (mean (SD))		38.73 (5.56)	41.82 (4.70)	** < 0.001**	41.66 (4.72)	38.46 (5.71)	**< 0.001**
ALB (%)	< 35	46 (22.9)	30 (8.9)	** < 0.001**	33 (8.9)	43 (25.7)	**< 0.001**
	≥ 35	155 (77.1)	308 (91.1)		339 (91.1)	124 (74.3)	
ALT (mean (SD))		59.95 (78.50)	52.96 (76.79)	0.312	52.03 (65.84)	63.44 (98.25)	0.114
ALT (%)	> 40	98 (48.8)	129 (38.2)	**0.02**	150 (40.3)	77 (46.1)	0.245
	≤ 40	103 (51.2)	209 (61.8)		222 (59.7)	90 (53.9)	
GGT (mean (SD))		128.36 (197.06)	110.22 (366.08)	0.517	116.04 (359.89)	119.09 (172.11)	0.917
GGT (%)	> 60	109 (54.2)	146 (43.2)	**0.017**	167 (44.9)	88 (52.7)	0.113
	≤ 60	92 (45.8)	192 (56.8)		205 (55.1)	79 (47.3)	
Size (mean (SD))		6.15 (3.59)	5.30 (3.26)	**0.005**	5.19 (3.15)	6.57 (3.77)	**< 0.001**
Number (%)	Single	152 (75.6)	271 (80.2)	0.256	290 (78.0)	133 (79.6)	0.744
	Multiple	49 (24.4)	67 (19.8)		82 (22.0)	34 (20.4)	
Stage (%)	I + II	138 (68.7)	265 (78.4)	**0.016**	293 (78.8)	110 (65.9)	**0.002**
	III + IV	63 (31.3)	73 (21.6)		79 (21.2)	57 (34.1)	
Grade (%)	1	10 (5.0)	21 (6.2)	0.368	23 (6.2)	8 (4.8)	0.054
	2	124 (61.7)	227 (67.2)		245 (65.9)	106 (63.5)	
	3	61 (30.3)	84 (24.9)		100 (26.9)	45 (26.9)	
	4	6 (3.0)	6 (1.8)		4 (1.1)	8 (4.8)	
Child (%)	5	120 (59.7)	246 (72.8)	**0.002**	276 (74.2)	90 (53.9)	**< 0.001**
	6	81 (40.3)	92 (27.2)		96 (25.8)	77 (46.1)	
Route (%)	conversion	29 (14.4)	36 (10.7)	0.062	45 (12.1)	20 (12.0)	**< 0.001**
	lapro	100 (49.8)	203 (60.1)		228 (61.3)	75 (44.9)	
	open	72 (35.8)	99 (29.3)		99 (26.6)	72 (43.1)	
Anatomical (%)	No	85 (42.3)	145 (42.9)	0.961	165 (44.4)	65 (38.9)	0.278
	Yes	116 (57.7)	193 (57.1)		207 (55.6)	102 (61.1)	
Major_resection (%)	No	131 (65.2)	245 (72.5)	0.091	270 (72.6)	106 (63.5)	**0.043**
	Yes	70 (34.8)	93 (27.5)		102 (27.4)	61 (36.5)	
Capsule (%)	complete	162 (80.6)	301 (89.1)	**0.009**	335 (90.1)	128 (76.6)	**< 0.001**
	InComplete	39 (19.4)	37 (10.9)		37 (9.9)	39 (23.4)	
Vascular_invasion (%)	No	148 (73.6)	268 (79.3)	0.159	287 (77.2)	129 (77.2)	1
	Yes	53 (26.4)	70 (20.7)		85 (22.8)	38 (22.8)	
MVI (%)	M0	122 (60.7)	220 (65.1)	0.32	242 (65.1)	100 (59.9)	0.441
	M1	42 (20.9)	72 (21.3)		77 (20.7)	37 (22.2)	
	M2	37 (18.4)	46 (13.6)		53 (14.2)	30 (18.0)	

*ALB* albumin, *ALT* alanine aminotransferase, *AFP* alpha-fetoprotein, *BMI* body mass index, *HDLC* high-density lipoprotein cholesterol, *TC* total cholesterol, *TG* triglycerides, *GGT* glutamyl transpeptidase, *HBsAg* hepatitis B surface antigen, *MVI* microvascular invasion

Bold P: *P* < 0.05

### Independent prognostic factors affecting OS

**Fig. 2:**
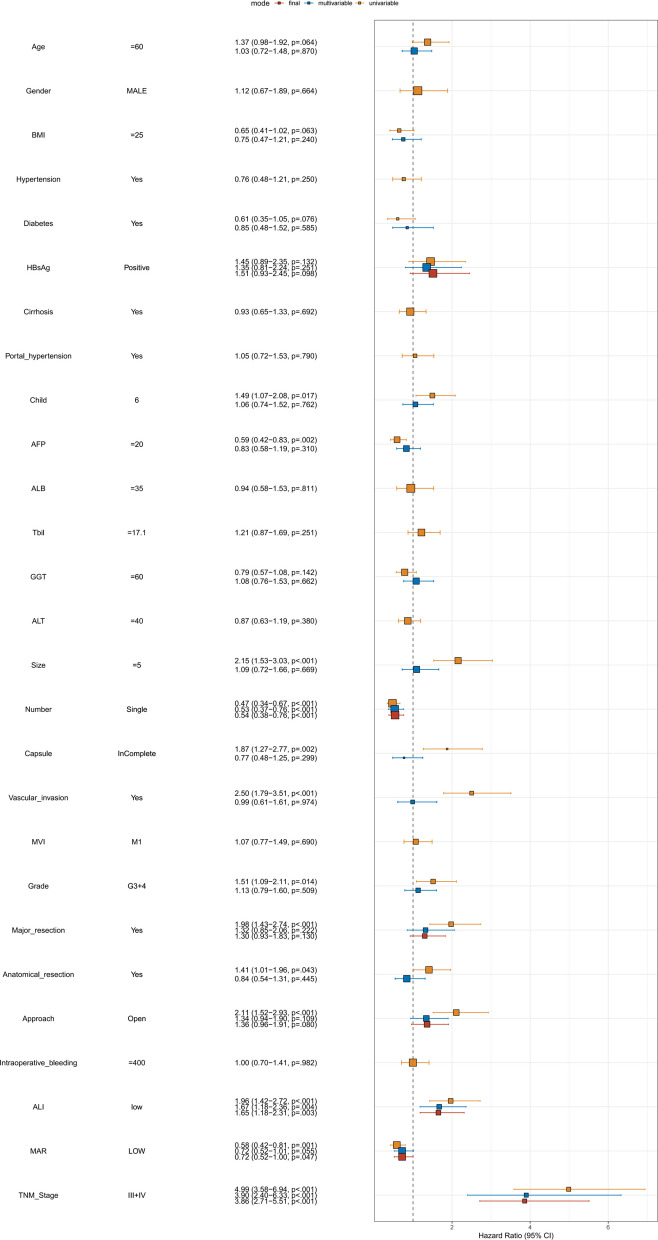
Forrest plot of the univariate and multivariate Cox regression analysis in HCC. BMI: body mass index; HBsAg: hepatitis B surface antigen; AFP: alpha-fetoprotein; ALB, albumin; ALT, alanine aminotransferase; Tbil, total bilirubin; GGT, glutamyl transpeptidase; ALT, alanine aminotransferase; MVI: microvascular invasion; TNM: tumor node metastasis classification. ALI: advanced lung cancer inflammation index; MAR: monocyte to albumin ratio

All recorded clinicopathological variables were first subjected to univariate Cox regression analysis. Covariates with a *p* value < 0.05 were then entered into a multivariable Cox proportional hazards model. In the multivariable analysis, TNM stage, tumor number, MAR, and ALI emerged as four independent prognostic indicators for OS (all *p* < 0.05) (Fig. 2 and Table 2).

**Table 2:** Cox proportional hazards regression model showing the association of variables with overall survival

Variable	Univariable		Multivariable	
	**HR(95%CI).x**	***p*-value**	**HR(95%CI).y**	***p*-value**
Factors selected				
AFP	1.451(1.144–1.839)	0.002	1.156(0.9–1.484)	0.256
Vascular_invasion	1.913(1.507–2.428)	< 0.001	0.962(0.689–1.341)	0.818
TNM_Stage	3.115(2.465–3.936)	< 0.001	2.638(1.879–3.702)	< 0.001
Size	1.721(1.351–2.191)	< 0.001	1.076(0.812–1.425)	0.612
Number	1.697(1.334–2.159)	< 0.001	1.587(1.235–2.04)	< 0.001
MAR	0.683(0.544–0.858)	0.001	0.787(0.624–0.993)	0.043
Major_resection	1.618(1.286–2.037)	< 0.001	1.189(0.872–1.621)	0.275
Grade	1.341(1.06–1.696)	0.014	1.112(0.868–1.423)	0.401
Child	1.329(1.052–1.677)	0.017	1.052(0.815–1.358)	0.695
Capsule	1.559(1.181–2.057)	0.002	0.823(0.592–1.145)	0.248
Approach	2.107(1.517–2.928)	< 0.001	1.356(0.955–1.924)	0.089
Anatomical_resection	1.274(1.007–1.611)	0.043	0.904(0.666–1.228)	0.519
ALI	1.964(1.418–2.72)	< 0.001	1.665(1.177–2.355)	0.004

We assessed the functional form of the association between continuous preoperative MAR and overall survival using restricted cubic splines. The analysis revealed no significant deviation from linearity (P for nonlinearity = 0.119; Supplementary Figure S1). The linear Cox model demonstrated comparable fit to the RCS model (ΔAIC = 1.8), supporting the treatment of MAR as a continuous linear variable in multivariable analysis. When modeled as a continuous linear term, each unit increase in MAR was associated with a hazard ratio of 1.002 (95% CI: 1.000–1.004, *P* = 0.048). These results indicate a monotonically increasing, approximately linear relationship between MAR and mortality risk.

### Kaplan–Meier survival analysis

**Fig. 3:**
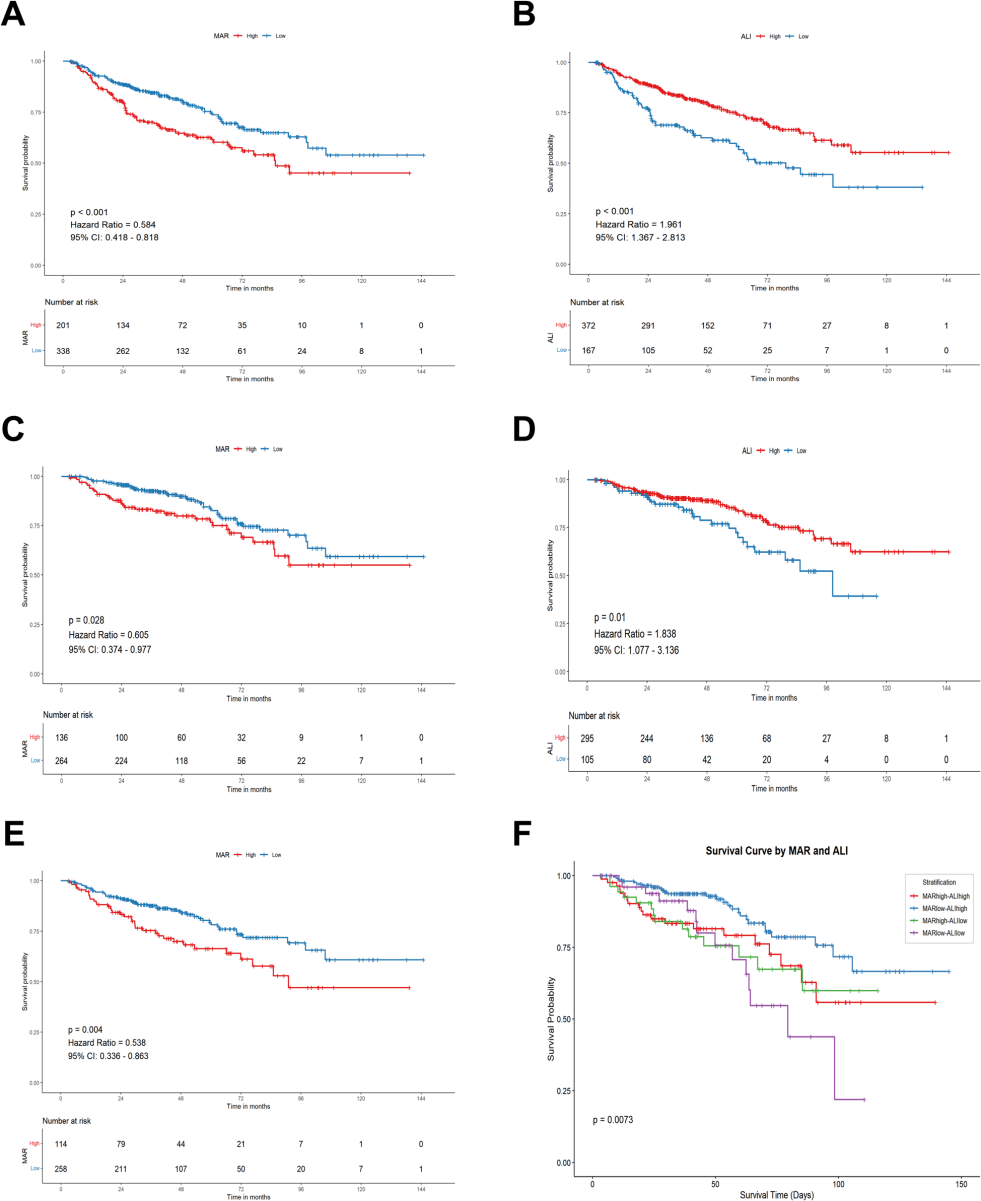
Kaplan–Meier survival curves comparing overall survival in patients stratified by MAR and ALI values. **A** OS for MAR-high vs. MAR-low groups. **B** OS for ALI-high vs. ALI-low groups. **C** OS for MAR-high vs. MAR-low groups for patients in TNM Stage I + II. **D** OS for ALI-high vs. ALI-low groups for patients in TNM Stage I + II. **E** OS for MAR-high vs. MAR-low groups for patients with high ALI value. **F** Kaplan–Meier survival analysis based on combined stratification of MAR and ALI. Patients were divided into four groups: MAR-high/ALI-high, MAR-low/ALI-high, MAR-high/ALI-low, and MAR-low/ALI-low

The impact of MAR and ALI on survival is summarized in Fig. 3. Patients with a low MAR had significantly longer OS than those with a high MAR (HR = 0.58, 95% CI: 0.42–0.82, *p* < 0.001; Fig. 3A). Likewise, a high ALI was strongly associated with improved survival compared with a low ALI (HR = 1.96, 95% CI: 1.37–2.81, *p* < 0.001; Fig. 3B). The favorable prognostic effect of low MAR was consistent in several stratified analyses. In the overall cohort (Fig. 3A), in patients with TNM stage I–II disease (Fig. 3C), and in the subgroup with high ALI values (Fig. 3E), low MAR was uniformly associated with better OS. Similarly, high ALI was linked to superior survival both in the entire cohort (Fig. 3B) and in relevant subgroups (Fig. 3D). Figure 3F depicts survival curves according to combinations of MAR and ALI categories, highlighting the joint influence of these two indices. Consistent with the above findings, patients with concurrent low MAR and high ALI had the most favorable prognosis after hepatectomy among all risk groups.

### Prognostic nomogram for OS

**Fig. 4:**
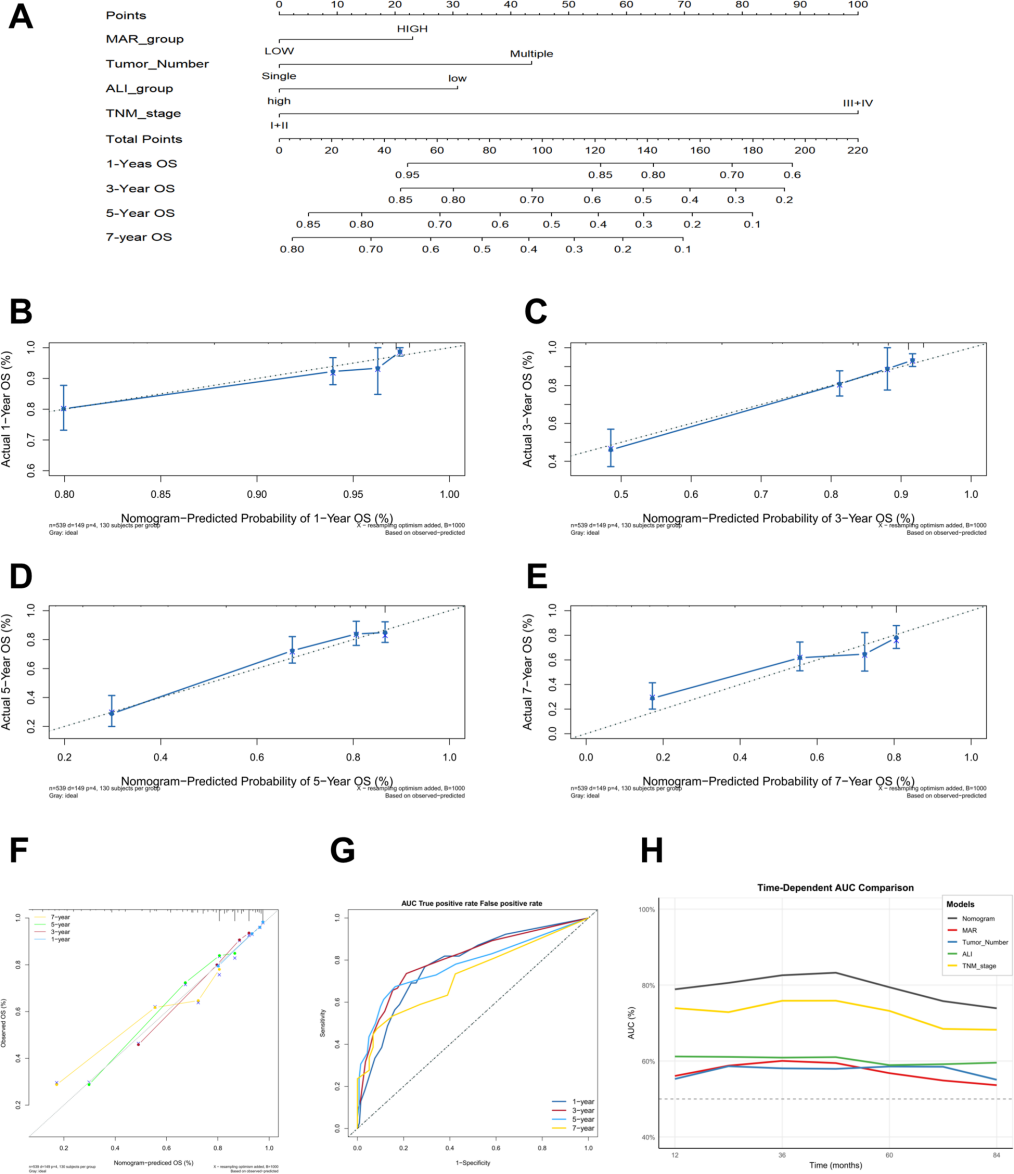
Development and validation of the progonostic nomogram. **A** The nomogram plot was built based on four independent prognostic factors in HCC. **B**-**E** Calibration curves of the nomogram. Calibration curves for 1-year (**B**), 3-year (**C**), 5-year (**D**), and 7-year (**E**) OS, respectively. **F** The calibration plot for internal validation of the nomogram. **G** The time-dependent AUC curves of the nomogram. **H** The time-dependent AUC curves of the nomogram

A prognostic nomogram was developed to estimate 1-, 3-, 5-, and 7-year OS (Fig. 4A). The nomogram incorporates four easily obtainable clinical parameters: MAR, ALI, tumor number, and TNM stage. For an individual patient, points are assigned for each variable and summed; the total score is then projected vertically onto the corresponding survival scale to derive the predicted probability of OS at each time point. The inclusion of ALI, MAR, TNM stage, and tumor number in the model further confirms that these factors are strong and independent determinants of postoperative survival in this HCC population.

### Model calibration and time-dependent discriminative ability

We rigorously examined the calibration of the nomogram both overall and at specific time points. The combined calibration plot (Fig. 4F) indicates close agreement between the probabilities predicted by the nomogram and the observed survival across all follow-up intervals. This overall pattern is consistent with the time‑specific calibration curves at 1, 3, 5, and 7 years (Fig. 4B–E). The bias‑corrected curves (black lines) are located very near the ideal 45-degree reference line (gray dashed lines), indicating good calibration. In particular, the calibration for 1‑ and 3‑year OS was excellent. Although the 7‑year predictions showed some underestimation in patients with lower predicted survival probabilities, the overall calibration of the model remained acceptable. The 5‑year OS curve (Fig. 4D), a commonly used endpoint in oncologic studies, demonstrated especially high accuracy. Bootstrap resampling (B = 1000) confirmed that the model was stable and not markedly overfitted.

The discriminative ability of the nomogram was evaluated using time‑dependent ROC curves. As depicted in Fig. 4G, the model achieved high AUC values for all examined time points: 0.78 for 1‑year OS, 0.81 for 3‑year OS, 0.79 for 5‑year OS, and 0.73 for 7‑year OS. All AUCs exceeded 0.7, indicating strong capacity to distinguish between patients with favorable and unfavorable outcomes. The ROC curves bending toward the upper left corner of the plots further reflect high sensitivity and specificity. The consistently good performance over a 7‑year period suggests that the nomogram has durable discriminative power for long‑term prognosis.The C-index of the nomogram also showed a consistent high compared to conventional TNM staging at all the time points that were assessed (Fig. 4H).

### Clinical utility and long-term accuracy of the prognostic model

**Fig. 5:**
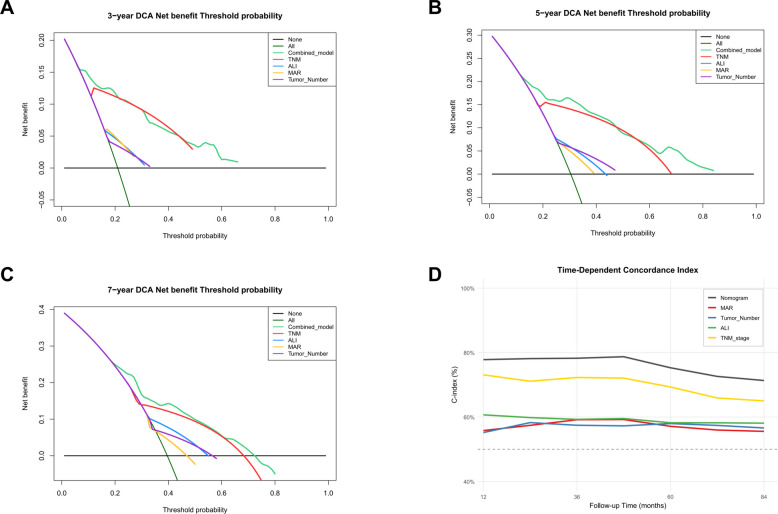
Prognostic model performance and clinical utility. **A**-**C** The probability of DCA net income threshold at different time nodes. **D** The time-dependent concordance index of the nomogram

Decision curve analysis was used to assess the clinical usefulness of the nomogram for predicting 3‑, 5‑, and 7‑year OS (Fig. 5A–C). Across a broad range of threshold probabilities, use of the nomogram provided a greater net benefit than strategies of treating all patients or treating none. These findings imply that applying the nomogram to identify high‑risk patients for intensified adjuvant therapy or closer surveillance, while sparing low‑risk individuals from unnecessary interventions, could offer meaningful clinical benefit. Within the first 3 years after surgery, the net benefit of the nomogram was similar to that of decisions based on TNM stage alone, whereas at 5 and 7 years the nomogram clearly outperformed TNM‑based strategies.

During the follow up, the nomogram gave larger AUC compared to TNM staging system (Fig. 5D) proving its better ability to distinguish between high and low risk patients. It is worth noting that the values of the nomogram of the AUC at 84 months of hepatectomy were still greater than 0.7, which highlights the strong predictive value of the nomogram in the long term. Its current edge over TNM staging emphasizes the value added to the conventional anatomical staging plans by the introduction of MAR and ALI.

### Subgroup analysis of the association between low value MAR and overall survival

**Fig. 6:**
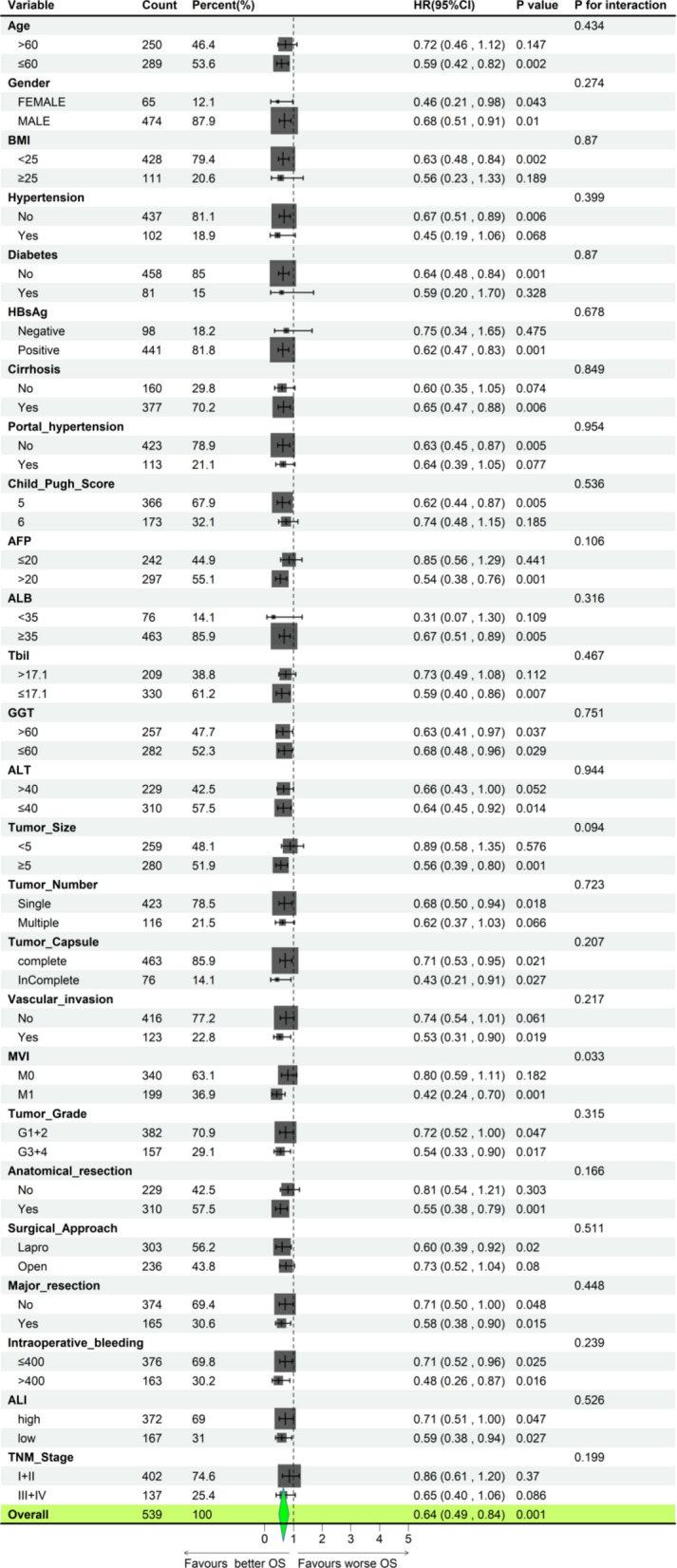
Forrest plot of the subgroup survival using univariate Cox regression analysis in HCC. ALB, albumin; ALT, alanine aminotransferase; AFP: alpha-fetoprotein; BMI: body mass index; GGT, glutamyl transpeptidase; HBsAg: hepatitis B surface antigen; MVI: microvascular invasion; TNM: tumor node metastasis classification; ALI: advanced lung cancer inflammation index

We also investigated the prognostic value of MAR in a given predetermined subgroups (Fig. 6). Low MAR was linked to a 32% risk of death decrease relative to the high MAR in the entire cohort (HR = 0.68, 95 percent CI: 0.54–0.86, *p* = 0. 001). These findings can visually be summarized in the forest plot with the square markers being the HR estimates of each of the subgroups, the horizontal lines representing the 95% CI of each subgroup, and the diamond representing the pooled HR and 95% CI of the entire cohort. Interaction *P* values were performed to test whether the MAR effect is heterogeneous in subgroups.

The survival benefit of low MAR was consistent and homogeneous across almost all predefined strata, including those based on age, sex, liver function (Child–Pugh score, presence of cirrhosis), tumor burden (tumor size, tumor number, vascular invasion), pathological features (histological grade, capsule status), and surgical factors (surgical approach and extent of resection). In nearly every subgroup, the HRs were < 1, favoring the low‑MAR group. Importantly, no significant interactions were detected between low MAR and any of the subgroup variables (all P for interaction > 0.05). These findings indicate that the prognostic advantage conferred by low MAR is robust and broadly applicable across diverse clinical and pathological backgrounds.

## Discussion

In this retrospective cohort study, we identified the preoperative MAR as a simple, independent, and clinically accessible prognostic factor for overall survival in patients with hepatocellular carcinoma undergoing curative hepatectomy. Patients with lower MAR exhibited significantly better survival outcomes, suggesting a more favorable host inflammatory and nutritional background. This prognostic significance persisted after comprehensive adjustment for established clinicopathological variables in multivariable analysis. To translate this finding into a practical tool, we developed a prognostic nomogram integrating MAR, the Advanced Lung Cancer Inflammation Index (ALI), tumor number, and TNM stage. This model demonstrated superior predictive performance and clinical utility compared to the traditional TNM staging system alone, providing an effective method for individualized risk assessment with potential for integration into clinical practice.

### Methodological considerations and the continuous risk gradient

One of the major aspects of real-world implementation is the question of whether MAR threshold determined in the given research could be generalized. Our single-center data statistically derived the optimum cut-off of 145.77 which may not be directly applicable to other centers or populations with similar baseline variables, laboratory platform, and treatment regimens. This cut-off as a standard reference in clinical guidelines should therefore not be adopted before it has been validated by external validation in large, multi-central cohorts.

To address methodological concerns regarding potential overfitting from dichotomization using a single, internally derived cut-off value, we performed supplementary analyses treating MAR as a continuous variable. Restricted cubic splines analysis confirmed a linear dose–response relationship between preoperative MAR and mortality risk (P for nonlinearity = 0.119), mitigating concerns about overoptimism associated with cut-off-based analyses. This linear association indicates that MAR reflects a graded biological risk rather than a threshold phenomenon. Although the per-unit hazard ratio appears modest, the interquartile range difference in our cohort (approximately 80 units) corresponds to a clinically meaningful 16% increase in mortality risk, an effect size comparable to other established prognostic indices in oncology. Notably, the prognostic independence of MAR highlights the value of the ratio over its individual components; while neither monocyte count nor albumin alone remained significant in our multivariable model, their ratio encapsulates a clinically pertinent imbalance between inflammation and nutritional synthesis.

### Biological plausibility: integrating inflammation and nutrition

The association between MAR and prognosis is biologically grounded in the interconnected pathways of systemic inflammation and nutritional status. Circulating monocytes are precursors to tumor-associated macrophages (TAMs), particularly the pro-tumorigenic M2 phenotype, which fosters tumor progression by promoting angiogenesis, suppressing anti-tumor immunity, and facilitating invasion and metastasis [15–21]. Conversely, albumin, a negative acute-phase protein, is modulated by both nutritional status and inflammatory cytokines such as IL-6 [22–24]. Consequently, a high MAR quintessentially represents a dual adverse condition of heightened inflammation and compromised nutrition, creating a microenvironment conducive to HCC progression. Our finding that ALI—another composite index reflecting inflammation, nutrition, and body composition—also emerged as an independent prognostic factor further underscores the critical role of the host’s inflammatory-nutritional axis in HCC outcomes. This aligns with the established prognostic value of indices like the Neutrophil-to-Lymphocyte Ratio (NLR) and Prognostic Nutritional Index (PNI) in various malignancies [25–30]. The concurrent significance of MAR and ALI, supported by a variance inflation factor < 2.0 (MAR is 1.329528, ALI is 1.139241) indicating no substantial collinearity, suggests they capture complementary dimensions of the host’s systemic response.

It is important to note that we did not make our study to elucidate the specific biological processes that associate MAR with the progression of HCC. However, despite the close implication of the role of monocytes, albumin, and inflammatory-nutritional condition, the detailed mechanism of TAM polarization, network of cytokines, and individual molecular pathways in HCC have not been totally clarified. To break-down the tumor biology of changes in MAR, further mechanistic work, such as in vitro work and in vivo models is required.

### Clinical translation and model utility

The primary clinical value of our findings lies in the developed nomogram, which synergistically combines routinely available host-based biomarkers (MAR, ALI) with tumor-centric features. This provides a more holistic “host-tumor” assessment than anatomical staging alone. The model demonstrated good calibration and maintained time-dependent AUCs above 0.7 over a 7-year period, indicating robust and sustained discriminative ability. Decision curve analysis confirmed its superior net clinical benefit across a wide range of threshold probabilities compared to strategies based solely on TNM staging.

Subgroup analyses give us further evidence of the strength of the overall finding. The relationship between low MAR and better OS was significant in all the preset subgroups and there was no significant interaction effects. That is, it did not seem that the survival department of low MAR was contingent upon age, sexuality, liver performance (including Child–Pugh classification and cirrhosis), tumor bulk (size, quantity, vascular invasion), pathology (grade, capsule status), or the surgical factors (approach and extent of resection). This consistent protective effect among different patient groups implies that MAR can be widely applied, and may be used in risk stratification programs of a large and diverse range of HCC patients.

This tool could aid in refining postoperative management in several ways: first, by identifying high-risk patients who may benefit from intensified surveillance or consideration for adjuvant therapy within clinical trials; second, by reassuringly stratifying low-risk patients who might be candidates for less intensive follow-up, thereby optimizing resource allocation. It is important to position this model as a potential complement to, rather than a replacement for, comprehensive staging systems like the BCLC classification, adding a quantifiable host-status dimension particularly within the surgically eligible patient subset.

### Study limitations and future perspectives

Our study has several limitations that must be acknowledged. First, the MAR cut-off value was statistically derived from this single-center cohort and requires external validation in multi-center, prospective studies before it can be recommended for generalized clinical use. Second, while we excluded patients with OS < 90 days (*n* = 8) and patients with with recurrence free survival < 90 days (*n* = 55) to minimize the impact of early postoperative non-cancer-related mortality (e.g., surgical complications, liver failure)—a common approach in oncologic studies—this may introduce a selection bias by underestimating the prognostic impact of MAR on very early survival, and thus our findings are most applicable to patients who survive the perioperative period. Third, although we adjusted for liver function (Child–Pugh score) and portal hypertension in our models and found them non-significant in the final multivariable analysis, residual confounding from these or other unmeasured factors may still exist. Fourth, our study did not elucidate the specific molecular mechanisms linking MAR to HCC progression. Future mechanistic studies are needed to dissect the related pathways of monocyte polarization and cytokine networks.

Interestingly, the inclusion of MAR and ALI in the multivariable model rendered some traditional pathological factors like vascular invasion non-significant. This suggests that the prognostic influence of such factors may be partly mediated through systemic host responses captured by these indices, further emphasizing the central role of the host environment. Ultimately, prospective, multi-center cohorts are required to validate the prognostic utility of MAR and our nomogram, and to explore its dynamic changes in relation to treatment response. The consistent protective association of low MAR across all prespecified subgroups in our study supports its broad potential applicability for risk-stratifying a diverse HCC population.

## Conclusion

Collectively, our results suggest that preoperative MAR is a good and valid prognostic factor of OS among patients having curative hepatectomy in the context of HCC. The use of MAR in perioperative assessment could assist clinicians to identify persons at high risk, establish more aggressive follow-up plans, and streamline the postoperative care. Multi-center, prospective studies in the future are justified to confirm the best MAR cut-off, in addition, to understand the underlying biological processes and whether there are any dynamic variations in MAR, which are associated with treatment, that may be used as an indicator of response to treatment and monitoring of the disease.

## Supplementary Information

Supplementary Material 1: Figure S1. Dose-response relationship between preoperative MAR and mortality risk. The solid blue line represents the adjusted hazard ratio (HR) with the shaded area indicating the 95% confidence interval. The red dashed line denotes the reference (HR = 1). The green dotted line indicates the median MAR value (123.8). The *P* value for nonlinearity was 0.119, supporting a linear association between continuous MAR and mortality risk after adjustment for tumor stage, tumor number, and ALI.

## Data Availability

The datasets generated and/or analyzed during the current study are not publicly available due to patient privacy and institutional regulations of Meizhou People’s Hospital, but are available from the corresponding author on reasonable request and with permission from the institutional ethics committee.

## Abbreviations

HCC: Hepatocellular carcinoma
MAR: Monocyte-to-albumin ratio
OS: Overall survival
C-index: Concordance index
ROC: Receiver operating characteristic
ALI: Advanced Lung Cancer Inflammation Index
STROBE: Strengthening the Reporting of Observational Studies in Epidemiology
BMI: Body mass index
AFP: Alpha-fetoprotein
MVI: Microvascular invasion
AJCC: American Joint Committee on Cancer
AUC: Area under the curve
DCA: Decision curve analysis
HR: Hazard ratio
IQR: Interquartile range
TNM: Tumor-node-metastasis
TAMs: Tumor-associated macrophages
IL-6: Interleukin-6
NLR: Neutrophil-to-lymphocyte ratio
PNI: Prognostic nutritional index
RCS: Restricted cubic splines
