# Supplementary material for: Preoperative monocyte-to-albumin ratio predicts overall survival after hepatectomy for hepatocellular carcinoma: a single-center retrospective study
Source: World J Surg Oncol. 2026 Jun 6;24:334. doi: 10.1186/s12957-026-04429-w (PMC13471592; doi:10.1186/s12957-026-04429-w)
Supplement: Supplementary file 1 [file 12957_2026_4429_MOESM1_ESM.docx]

**Figure.S1** Dose-response relationship between preoperative MAR and mortality risk.

The solid blue line represents the adjusted hazard ratio (HR) with the shaded area indicating the 95% confidence interval. The red dashed line denotes the reference (HR = 1). The green dotted line indicates the median MAR value (123.8). The P value for nonlinearity was 0.119, supporting a linear association between continuous MAR and mortality risk after adjustment for tumor stage, tumor number, and ALI.
